# Outbreak of Porcine Reproductive and Respiratory Syndrome Virus 1 in Taiwan

**DOI:** 10.3390/v12030316

**Published:** 2020-03-16

**Authors:** Wei-Hao Lin, Kraijak Kaewprom, Sheng-Yuan Wang, Chuen-Fu Lin, Cheng-Yao Yang, Ming-Tang Chiou, Chao-Nan Lin

**Affiliations:** 1 Animal Disease Diagnostic Center, College of Veterinary Medicine, National Pingtung University of Science and Technology, Pingtung 91201, Taiwan; j7967633@gmail.com (W.-H.L.); april89000@gmail.com (S.-Y.W.); 2 Department of Veterinary Medicine, College of Veterinary Medicine, National Pingtung University of Science and Technology, Pingtung 91201, Taiwan; addyvm.rmu@gmail.com; 3 Program of Veterinary Technology, Faculty of Agricultural Technology, Rajabhat Mahasarakham University, Mahasarakham 44000, Thailand; 4 Department of Veterinary Medicine, College of Veterinary Medicine, National Chiayi University, Chiayi 60054, Taiwan; linchuenfu@gmail.com; 5 Graduate Institute of Veterinary Pathobiology, College of Veterinary Medicine, National Chung Hsing University, Taichung 40227, Taiwan; yangchengyao@nchu.edu.tw

**Keywords:** porcine reproductive and respiratory syndrome virus 1, European type, Taiwan

## Abstract

Porcine reproductive and respiratory syndrome (PRRS) causes significant economic losses in the swine industry worldwide. The PRRS virus (PRRSV) can be divided into two species, PRRSV 1 (European) and PRRSV 2 (North American). In Taiwan, PRRSV 2 isolates are dominant and cause respiratory symptoms in nursing pigs. From October to November 2018, in a pig herd in central Taiwan, pregnant sows had abortions and stillbirths, and piglets suffered from respiratory disorders. Laboratory tests identified the presence of PRRSV 1 in serum from sows and suckling piglets in this scenario. The complete genome of the identified PRRSV 1 strain was genetically closely related to that of a European PRRSV vaccine strain (98.2%). This local European isolate is designated as PRRSV/NPUST-2789-3W-2/TW/2018 (NPUST2789). This report is the first to indicate an outbreak in Taiwan of a PRRSV 1 strain that shares a common evolutionary ancestor with the European PRRSV vaccine strain.

## 1. Introduction

Porcine reproductive and respiratory syndrome (PRRS) is one of the most economically devastating diseases facing the pork industry worldwide. The causative agent, the PRRS virus (PRRSV), was identified in the early 1990s [1,2]. A retrospective analysis revealed the presence of PRRSV antibodies in Canadian samples as early as 1979 [3]. PRRSV is an enveloped, positive-stranded RNA virus with a genome measuring approximately 15 kb. Recently, PRRSV was reclassified to the genus *Betaarterivirus* and belongs to the family Arteriviridae within the order Nidovirales, which is divided into two species, *Betaarterivirus suid 1* (PRRSV 1, European) and *Betaarterivirus suid 2* (PRRSV 2, North American) [4]. There is approximately 60% genomic sequence identity between the PRRSV 1 and PRRSV 2 strains [5,6,7]. 

During PRRSV infection, clinical disease is detectable in pigs of all of ages [8]. The clinical presentation of PRRSV can range from asymptomatic to devastating, with symptoms such as anorexia, high fever, hyperpnea, abortion, stillbirth or death in sows [8]. Suckling piglets are often infected by PRRSV through vertical or horizontal transmission. PRRSV infection in suckling piglets is characterized most consistently by listlessness, emaciation, splay leg posture, hyperpnea, dyspnea, and increased preweaning mortality [8]. Although more than 30 years have passed since PRRSV was first described, current management strategies mainly focus on preventing PRRSV infection using vaccination [9]. However, the currently available modified live vaccines (MLVs) are not sufficient to eliminate the virus [9]. The MLV vaccine can elicit not only humoral but also cell-medicated immune responses against PRRSV. In addition to efficacy, reverse virulence of MLV [10], recombination between wild-type PRRS and MLV [11,12,13] or recombination between different MLVs had been reported [14,15]. Therefore, the safety and cross-protection of MLV warrant further investigation.

In Asia, PRRSV 1 and PRRSV 2 have been reported to exist in several countries, including China, Japan, South Korea, Thailand and Vietnam [16,17,18,19,20,21,22]. In Taiwan, PRRSV 2 is dominant, while PRRSV 1 still exists but rarely causes significant economic losses [23]. In the present study, we investigated the previously unidentified PRRSV 1 outbreak in Taiwan. We reported a genetic and phylogenetic analysis of the PRRSV 1 isolate, designated PRRSV/NPUST-2789-3W-2/TW/2018 (NPUST2789), which is genetically closely related to that of a European PRRSV vaccine strain.

## 2. Materials and Methods 

### 2.1. Clinical Cases

From October to November 2018, pregnant sows had abortions and stillbirths, and piglets suffered from respiratory disorders in a 550-sow farrow-to-finish pig farm located in central Taiwan. The farm has good external biosecurity and is free of porcine epidemic diarrheal virus. Neither sows nor piglets exhibited clinical signs of PRRS without PRRSV vaccination in this farm, which had been classified as positive stable and monitored by the Animal Disease Diagnostic Center (ADDC), National Pingtung University of Science of Technology (NPUST) [24].

### 2.2. Sample Preparation and Quantification of PRRSV RNA

Blood samples were collected from the affected animals and submitted to the ADDC, NPUST. Total nucleic acids were extracted from serum samples using a MagNA Pure LC total nucleic acid isolation kit (Roche Diagnostics GmbH, Mannheim, Germany) operated on a MagNA Pure 24 System (Roche Applied Science, Rotkreuz, Switzerland) according to the manufacturer’s protocol. All samples were tested for PRRSV by a modified PRRSV real-time PCR assay [25]. The primers PRRSV-M177F (5′-CATTCTGGCCCCTGCCCA-3′), PRRSV M177R (5′-ACCACTCCYYGYTTDACAGCT-3′), the PRRSV 1 zip nucleic acid (ZNA) probe (HEX-CGCTGTGAGAAAGCCCGG-ZNA4 BHQ1) and PRRSV 2 ZNA probe (FAM-CTCGTGTTGGGTGGCAGA-ZNA4 BHQ1), which targets the M gene, were used for quantitative real-time polymerase chain reaction to detect PRRSV in the clinical specimens.

### 2.3. Serology

All serum samples were tested for the presence of anti-PRRSV antibodies using a commercial ELISA kit (IDEXX PRRS X3 Ab Test; IDEXX Laboratories Inc, Westbrook, ME, USA) according to the manufacturer’s instructions. Serum samples were considered positive for the presence of PRRSV antibody when the average sample-to-positive (S/P) ratio was ≥ 0.4.

### 2.4. Virus Isolation and Whole Genome Sequencing of PRRSV

To harvest the highest viral load for whole genome sequencing of PRRSV, 0.5 mL of PRRSV-positive serum was added to Meat Animal Research Center-145 (MARC-145) cells and observed daily for cytopathic effects. Nucleic acids were extracted from the culture medium using a MagNA Pure 24 System (Roche Applied Science, Rotkreuz, Switzerland). The nucleic acids were end-repaired, A-tailed and adapter-ligated following the Illumina TruSeq DNA preparation protocol. After library construction, it was used for next-generation sequencing (NGS) by a NovaSeq 6000 System (Illumina, San Diego, CA, USA).

### 2.5. Open Reading Frame 5 Amplification and Sequencing

The open reading frame 5 (ORF5) genes were amplified and sequenced as described in a previous study [26]. The nucleotide sequences were determined from both orientations by an ABI 3730XL DNA analyzer (Applied Biosystems, Foster City, CA).

### 2.6. Sequence and Phylogenetic Analysis

The complete sequences of local PRRSV were then compared with other reference PRRSV strains, and the results are summarized in Table 1. Multiple alignments of nucleic acid sequences were performed by the Clustal W methods in Molecular Evolutionary Genetics Analysis X software (MEGA X) [27]. Phylogenetic trees were constructed using the maximum likelihood method based on the Kimura 2-parameter model in MEGA X [27]. The sequence identity of nucleotides and amino acids was determined by MegAlign software (Lasergene, DNASTAR, Madison, WI, USA). The similarity versus position was plotted by SimPlot v3.5.1 software within a 200-bp window sliding along the genome alignment (20-bp step size) [28].

## 3. Results

### 3.1. Emergence of PRRSV 1

**Figure 1:**
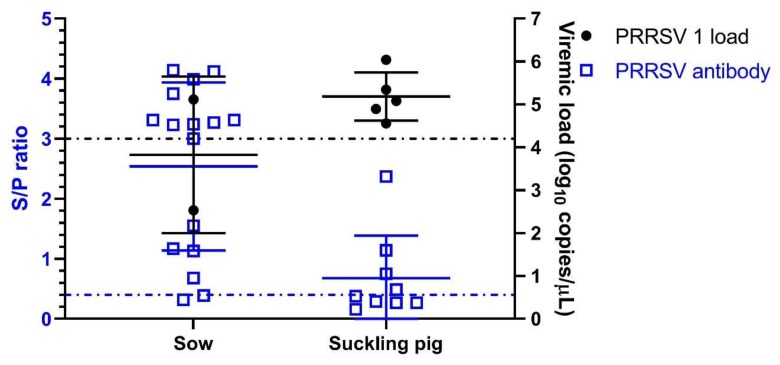
PRRSV 1 load and antibody level (sample-to-positive (S/P) ratio) of sows and suckling piglets suffered from porcine reproductive and respiratory syndrome (PRRS) in the present study.

A total of 70 sows were culled due to this outbreak, including 50 aborted sows, 5 sudden deaths of sows and 15 sows with severe anorexia. The suckling piglets were unthrifty and exhibited dyspnea and increased the preweaning mortality in this period. Forty percent of weaned pigs showed severe respiratory signs and 25% mortality among the nursery piglets. In laboratory testing, 3 of 16 affected sows (18.75%, viral load range from 1 × 10^1^ to 1.3 × 10^5^ genomes/µL) and 5 of 9 affected suckling piglets (55.6%, viral load range from 3.6 × 10^4^ to 1.1 × 10^6^ genomes/µL) were positive for PRRSV 1 (Figure 1). The average sample-to-positive (S/P) ratio of the PRRSV antibody of these sows was 2.54, ranging from 0.32 to 4.14, and the positive rate was 87.5%. Ten of 16 sows had S/P ratios higher than 3.0, as determined using a commercial ELISA kit (Figure 1). Collectively, this farm suffered a PRRSV 1 outbreak, and this scenario is the first in Taiwan to be reported.

### 3.2. Phylogenetic Analysis of Whole Genome Sequences

**Figure 2:**
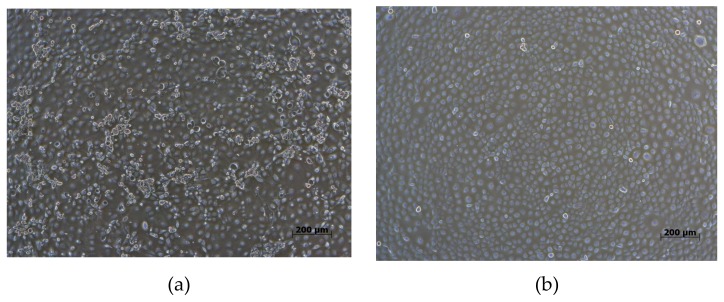
(**a**) Meat Animal Research Center-145 (MARC-145) cells cultured with serum from diseased suckling piglets. (**b**) Uninfected MARC-145 cells.

**Figure 3:**
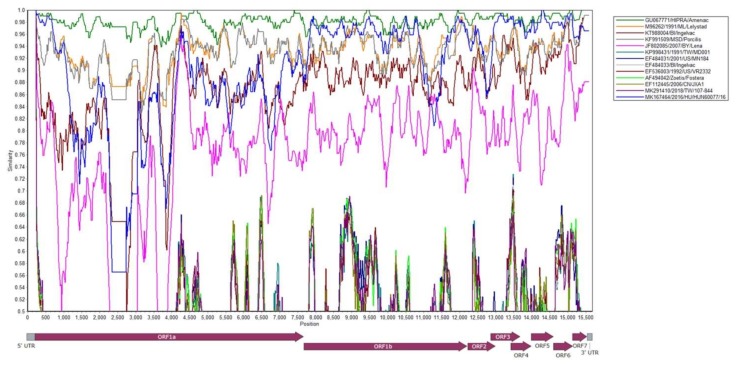
Nucleotide sequence similarity of the complete genome of PRRSV/NPUST-2789-3W-2/TW/2018 with that of other PRRSVs.

**Figure 4:**
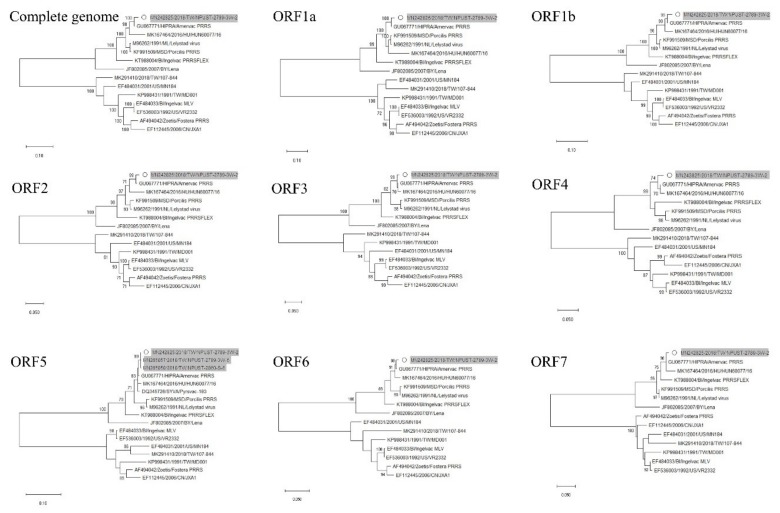
Phylogenetic relationships constructed using complete genomes, ORF1a, ORF1b, ORF2, ORF3, ORF4, ORF5, ORF6 and ORF7 gene sequences of PRRSV/NPUST-2789-3W-2/TW/2018 (NPUST2789) and other PRRSVs. The phylogenetic tree was constructed using the maximum likelihood method with bootstrap analysis (*n* = 1000) to evaluate clade confidence. Bootstrap support values greater than 70 are shown. The circle represents the PRRSV strain NPUST2789. The gray underlay represents local PRRSV 1 strains isolated in the present study.

Viremic serum from a 3-week-old piglet (NPUST-2789-3W-2) was used for PRRSV isolation by Meat Animal Research Center-145 (MARC-145) cells (Figure 2). This isolate was denominated as PRRSV/NPUST-2789-3W-2/TW/2018 (NPUST2789). The full genomic RNA sequence of NPUST2789 (accession number: MN242825) comprises 15,111 nucleotides, excluding the 3′ polyadenylated nucleotides. Similarity comparisons with SimPlot v3.5.1 revealed that the whole genome of NPUST2789 was most similar to that of the European PRRS vaccine Amervac PRRS (Figure 3). The entire genome of NPUST2789 was 98.2% and 94.1% similar to the European PRRS vaccine Amervac PRRS and prototype PRRSV 1 Lelystad virus, respectively (Table 1 and Figure S1). Phylogenetic trees based on the whole genomes and ORF 1a to ORF 7 nucleotide sequences showed the complete genome, and each ORF of NPUST2789 was most closely related to those of the known Amervac PRRS (Figure 4 and Figure S1).

### 3.3. Phylogenetic, Amino Acid and Nucleotide Sequence Analysis of ORF5

The ORF5 sequences from another 3-week-old piglet (NPUST-2789-3W-5, MN265856) and a viremic sow (NPUST-2860-S-6, MN265858) were almost identical to those of NPUST2789 (Figure 4, Table 1 and Figure S2). In contrast to the low nucleotide and amino acid sequence identities between other European PRRSV1 strains (Leylstad and HUN60077/16 strains) and Taiwanese PRRSV 1 NPUST2789, the sequence identity levels between this outbreak of PRRSV 1 isolates and Amervac PRRS appeared to be considerably higher (Table 2 and Figure S2). Taken together, these results confirmed that this outbreak of PRRSV 1 shared a common origin with Amervac PRRS.

## 4. Discussion

In the present study, we characterized a previously unidentified PRRSV 1 outbreak in Taiwan. A comparison of the data obtained was made with available sequences of entire PRRSV 1. This comparison clearly indicated a close relationship between our PRRSV 1 and the vaccine virus Amervac PRRS and less close relationship to other PRRSV1 vaccines (Table 1). To date, there are four commercial PRRSV MLV vaccines available in Taiwan, including Ingelvac PRRS^®^ MLV (PRRSV 2, Boehringer Ingelheim), Fostera^®^ PRRS (PRRSV 2, Zoetis), Amervac PRRS (PRRSV 1, Hipra) and Pyrsvac-183 PRRS (PRRSV 1, Syva) (Table 1). Interestingly, for the last decade, this herd has not been immunized with any PRRSV 1 or PRRSV 2 vaccine, and the farmer has never introduced boars or gilts from domestic breeding herds. The Amervac PRRS MLV vaccine has been used in Taiwanese pig farms for almost ten years. Therefore, we speculated that PRRSV 1 transmits into pig farms by the adaptation of Amervac PRRS MLV. A similar finding had been observed in PRRSV 2 in 1998; PRRSV 2 in Danish pig farms was due to the spread of the vaccine virus [29]. Therefore, the prevalence of PRRSV 1 in Taiwan warrants further investigation.

PRRSV 1 vaccine-like cases have been reported recently in Hungary [11], Denmark [15] and China [30]. The genome of the Hungarian PRRSV 1 strain HUN60077/16, which has a mosaic structure of the genome, showed a notably close genetic relationship to the PRRSV 1 vaccine Unistrain, also known as Amervac PRRS in Taiwan or China. A similar report was also found in China; Amervac-like isolates carried out the genes from vaccine strains and circulating wild strains [30]. Certain issues need to be further investigated: (i) other PRRSV 1 or PRRSV 2 vaccine-like cases; (ii) reversion to virulence of attenuated vaccine strains; and (iii) low-virulence strains causing outbreaks in PRRSV-stable or PRRSV-free farms. Therefore, the viral evolution, origin and the difference between vaccine and field strains warrant further investigation.

In conclusion, this report is the first to describe an outbreak in Taiwan of a PRRSV 1 strain that shared a common evolutionary ancestor with a European PRRSV vaccine strain. This PRRSV 1 outbreak in Taiwan was attributable to the spread of vaccine viruses.

## Figures and Tables

**Table 1:** Sequence identity of PRRSV/NPUST-2789-3W-2/TW/2018 and the reference strains.

Virus	Species	Year	Country/Company	Sequence Identity (%)		GenBank Accession Number
				Whole Genome	ORF5	
Amervac PRRS ^1^	PRRSV 1	N/A	HIPRA	98.2	99.0	GU067771
Porcilis PRRS	PRRSV 1	N/A	MSD	93.8	94.2	KF991509
Ingelvac PRRSFLEX	PRRSV 1	N/A	Boehringer Ingelheim	87.4	92.6	KT988004
Pyrsvac-183 ^1^	PRRSV 1	N/A	SYVA	N/A	98.5	DQ345726
Ingelvac PRRS ^2^	PRRSV 2	N/A	Boehringer Ingelheim	57.8	59.7	EF484033
Fostera PRRS ^2^	PRRSV 2	N/A	Zoetis	57.5	59.2	AF494042
Lelystad virus	PRRSV 1	1991	Netherlands	94.1	94.5	M96262
Lena	PRRSV 1	2007	Belarus	80.2	82.8	JF802085
HUN60077/16	PRRSV 1	2016	Hungary	91.7	98.2	MK167464
NPUST-2789-3W-2	PRRSV 1	2018	Taiwan	100	100	MN242825
NPUST-2789-3W-5	PRRSV 1	2018	Taiwan	N/A	99.8	MN265857
NPUST-2860-S-6	PRRSV 1	2018	Taiwan	N/A	99.8	MN265858
VR2332	PRRSV 2	1992	USA	57	59.9	EF536003
MD001	PRRSV 2	1991	Taiwan	56.1	58.4	KP998431
MN184	PRRSV 2	2001	USA	57.9	58.4	EF484031
JXA1	PRRSV 2	2006	China	57.3	60.7	EF112445
107-844	PRRSV 2	2018	Taiwan	57.8	56.8	MK291410

^1^ Modified live attenuated PRRSV 1 vaccines were used in Taiwan. ^2^ Modified live attenuated PRRSV 2 vaccines were used in Taiwan.

**Table 2:** Sequence identities of nucleotide and amino acid sequences between PRRSV/NPUST-2789-3W-2/TW/2018 and the reference strains.

ORF	Amervac PRRS		Lelystad Virus		HUN60077/16	
	Nucleotide (%)	Amino Acid (%)	Nucleotide (%)	Amino Acid (%)	Nucleotide (%)	Amino Acid (%)
ORF1a	98.1	98.1	93.6	94.4	87.2	87.6
ORF1b	98.2	99.1	94.3	98.2	95.4	98.6
ORF2	98.7	97.7	94.9	94.6	93.6	94.6
ORF3	98.9	99.6	94.5	94.4	98	98.5
ORF4	97.8	96.8	93.3	94.1	96.9	94.6
ORF5	99.8	100	98.5	97.1	98.2	97.1
ORF6	99.2	98.3	96.4	96.6	98.1	97.1
ORF7	99.2	98.5	96.2	96.2	96.7	96.2

## Supplementary Materials

The following are available online at https://www.mdpi.com/1999-4915/12/3/316/s1, Figure S1: Nucleotide sequence identity of the complete genome of PRRSV/NPUST-2789-3W-2/TW/2018 with that of other PRRSVs., Figure S2: Nucleotide sequence identity of ORF5 of PRRSV/NPUST-2789-3W-2/TW/2018 with that of other PRRSVs.
