# Supplementary material for: Outbreak of Porcine Reproductive and Respiratory Syndrome Virus 1 in Taiwan
Source: Viruses. 2020 Mar 16;12(3):316. doi: 10.3390/v12030316 (PMC7150920; doi:10.3390/v12030316)
Supplement: Supplementary file 1 [file viruses-12-00316-s001.pdf]

Percent Identity

Divergence

	1	2	3	4	5	6	7	8	9	10	11	12	13	14		
1		98.2	94.1	87.4	93.8	80.2	56.1	57.9	57.8	57.0	57.5	57.3	57.8	91.7	1	NPUST-2789-3W-2
2	1.8		95.1	87.9	94.8	80.6	56.4	58.0	58.0	57.2	57.6	57.3	57.8	92.6	2	Amervac-PRRS
3	6.5	5.2		88.6	98.9	80.5	56.2	57.8	57.6	56.8	57.5	57.2	57.6	91.8	3	Lelystad-virus
4	12.9	12.3	11.3		88.3	79.4	56.8	58.7	57.4	57.4	57.3	56.8	58.2	85.7	4	Ingelvac-PRRSFLEX
5	6.6	5.4	1.0	11.5		80.3	56.1	57.6	57.6	56.8	57.7	57.3	57.5	91.5	5	Porcilis-PRRS
6	24.1	23.7	23.6	24.9	23.8		55.8	58.0	57.3	56.6	57.1	56.9	58.0	79.4	6	Lena
7	58.7	58.3	58.5	57.8	58.6	58.9		81.9	87.0	88.4	85.8	84.0	79.1	56.0	7	MD001
8	57.8	57.7	58.2	57.6	58.2	58.4	15.8		84.9	83.4	83.8	82.5	88.1	57.7	8	MN184
9	58.1	57.9	58.5	57.9	58.3	58.4	12.0	14.9		98.1	91.2	89.0	82.0	57.5	9	Ingelvac-MLV
10	58.1	57.8	58.5	57.6	58.3	58.2	12.1	15.2	0.5		89.6	87.5	80.7	56.7	10	VR2332
11	58.5	58.1	58.6	57.6	58.4	58.5	13.0	16.0	9.2	9.5		91.7	81.8	57.4	11	Fostera-PRRS
12	58.7	58.6	59.1	58.5	58.8	58.6	15.0	17.2	11.5	11.8	8.1		80.7	56.9	12	JXA1
13	58.2	57.9	58.6	58.6	58.7	57.9	19.9	13.8	18.7	18.9	19.0	20.0		57.3	13	107-844
14	9.1	8.1	9.0	15.1	9.1	25.4	59.2	58.3	59.0	58.9	58.6	59.4	59.1		14	HUN60077-16
	1	2	3	4	5	6	7	8	9	10	11	12	13	14		

Figure S1. Nucleotide sequence identity of the complete genome of PRRSV/NPUST-2789-3W-2/TW/2018 with that of other PRRSVs.

Percent Identity

Divergence

	1	2	3	4	5	6	7	8	9	10	11	12	13	14	15	16	17		
1		99.8	99.8	99.0	98.5	94.2	92.6	94.5	82.8	98.2	59.7	59.2	59.9	60.7	58.4	58.4	56.8	1	NPUST-2789-3W-2
2	0.2		99.7	98.9	98.4	94.0	92.4	94.3	82.7	98.1	59.9	59.4	60.0	60.8	58.6	58.3	57.0	2	NPUST-2789-3W-5
3	0.2	0.3		98.9	98.4	94.0	92.4	94.3	82.7	98.1	59.5	59.1	59.7	60.5	58.3	58.3	56.6	3	NPUST-2860-S-6
4	1.0	1.2	1.2		99.2	94.3	92.6	94.8	82.7	98.5	60.2	59.7	60.4	60.5	58.9	58.7	57.3	4	Amervac-PRRS
5	1.5	1.7	1.7	0.8		94.2	92.4	94.7	82.4	98.4	60.4	59.9	60.5	60.7	59.1	58.9	57.4	5	Pyrsvac-183
6	6.3	6.5	6.5	6.1	6.3		89.8	98.9	83.3	94.2	60.5	60.7	60.7	60.4	58.9	59.2	58.1	6	Porcilis-PRRS
7	8.2	8.4	8.4	8.2	8.4	11.5		90.6	82.8	92.4	60.2	60.4	60.4	61.3	60.7	59.5	58.6	7	Ingelvac-PRRSFLEX
8	5.9	6.1	6.1	5.5	5.7	1.2	10.5		83.2	94.7	60.4	60.5	60.5	60.2	58.7	58.7	57.9	8	Lelystad-virus
9	20.6	20.9	20.9	20.9	21.4	19.9	20.6	20.1		82.7	59.1	58.3	59.5	58.7	57.8	57.6	58.3	9	Lena
10	1.8	2.0	2.0	1.5	1.7	6.3	8.4	5.7	20.9		59.5	59.1	59.7	60.2	58.3	58.1	56.6	10	HUN60077-16
11	53.0	52.6	53.4	51.9	51.5	51.3	52.1	51.7	54.3	53.5		91.6	99.4	89.2	86.9	87.1	88.0	11	Ingelvac-MLV
12	54.0	53.6	54.4	52.9	52.5	50.8	51.6	51.2	56.4	54.5	9.3		91.4	94.5	87.7	90.3	87.9	12	Fostera-PRRS
13	52.6	52.2	53.0	51.5	51.2	50.9	51.7	51.3	53.2	53.2	0.7	9.5		89.2	86.9	87.1	88.3	13	VR2332
14	50.5	50.2	50.9	50.9	50.6	51.4	49.3	51.8	55.0	51.7	12.2	5.9	12.2		87.5	88.5	87.1	14	JXA1
15	56.1	55.7	56.6	55.0	54.6	55.2	51.0	55.6	57.8	56.6	15.3	14.2	15.3	14.3		86.9	90.1	15	MN184
16	56.1	56.6	56.6	55.4	55.0	54.4	53.7	55.6	57.9	57.2	15.0	10.9	15.0	13.1	15.2		85.3	16	MD001
17	60.2	59.8	60.7	59.0	58.6	57.1	55.9	57.5	56.4	60.7	13.8	14.0	13.3	15.0	11.2	17.5		17	107-844
	1	2	3	4	5	6	7	8	9	10	11	12	13	14	15	16	17		

Figure S2. Nucleotide sequence identity of ORF5 of PRRSV/NPUST-2789-3W-2/TW/2018 with that of other PRRSVs.
